# Nepetin inhibits osteoclastogenesis by inhibiting RANKL‐induced activation of NF‐κB and MAPK signalling pathway, and autophagy

**DOI:** 10.1111/jcmm.16055

**Published:** 2020-11-01

**Authors:** Binxiang Chu, Shenao Chen, Xiaohe Zheng, Jiajing Ye, Xu Cheng, Liwei Zhang, Di Guo, Peng Wang, Dun Hong, Zhenghua Hong

**Affiliations:** ^1^ Department of Orthopedic Taizhou Hospital of Zhejiang Province Affiliated to Wenzhou Medical University Linhai China; ^2^ Department of Orthopedic Dajiangdong Hospital Hangzhou China; ^3^ Department of Pathology Taizhou Hospital of Zhejiang Province Affiliated to Wenzhou Medical University Linhai China

**Keywords:** bone destruction, nepetin, osteoclasts, TRAF6

## Abstract

Aseptic prosthetic loosening due to wear particle–induced inflammatory osteolysis is the main cause of failure for artificial joint replacement. The inflammatory response and the production of pro‐osteoclastic factors lead to elevation of osteoclast formation and excessive activity results in extensive bone destruction around the bone‐implant interface. Here we showed that Nepetin, a natural bioactive flavonoid with proven anti‐inflammatory and anti‐proliferative properties, potently inhibited RANKL‐induced osteoclast differentiation, formation and bone resorption in vitro, and protected mice against the deleterious effects of titanium particle–induced calvarial osteolysis in vivo. Mechanistically, Nepetin attenuated RANKL‐induced activation of NF‐κB and MAPK signalling pathways and TRAF6‐dependent ubiquitination of Beclin 1 which is necessary for the induction of autophagy. In brief, our study demonstrates the potential therapeutic application of Nepetin against osteoclast‐mediated osteolytic diseases.

## 1 INTRODUCTION

Joint replacement is a common surgical procedure for the terminal stages of degenerative joint diseases including rheumatoid arthritis and osteoarthritis.^1^ Despite advances in surgical procedures and prosthetic material, peri‐prosthetic osteolysis and the resulting aseptic loosening remains a major complication leading to implant failure.^2^ Titanium (Ti)‐based biomaterials are by far the most commonly used prosthetic implants due to their superiority in load‐bearing applications and excellent mechanical strength and resilience.^3^ However, it has now been suggested that wear particles derived from the metal biomaterial provoke an inflammatory response that subsequently induces osteoclast‐mediated bone destruction around the bone‐implant interface.^4^ The underlying molecular mechanism that bridges the initial inflammatory response to subsequent bone destruction is complex but involves the production and release of chemokines and cytokines that predominantly promote osteoclast recruitment and activity causing localized bone loss.^5^

Osteoclasts are multinucleated giant cells that originated from the monocyte/macrophage system.^6^ Osteoclasts are the unique cell in the body capable of bone resorption and hence most if not all osteolytic bone conditions are due to excessive osteoclasts activity and/or formation.^7^ Two key cytokines that dictate osteoclast differentiation and are released by inflammatory cells are receptor activator of nuclear‐κB ligand (RANKL) and macrophage‐colony‐stimulating factor (M‐CSF).^8^, ^9^ M‐CSF is required for osteoclast precursor survival and proliferation and up‐regulated expression of surface RANK, the cognate receptor for RANKL.^10^, ^11^ The combination of RANKL and RANK activates a series of signalling cascades, via TNF receptor‐associated factor (TRAF), the intracellular adaptor proteins. Activation of mitogen‐activated protein kinase (MAPK) and nuclear factor‐κB (NF‐κB) signalling pathways that are necessary for osteoclast differentiation and function occurs via TRAF2, TRAF5 and TRAF6, with TRAF6 playing an essential role in RANKL‐RANK signalling.^12^ The genetic loss of TRAF6 leads to osteopetrosis phenotype in mice highlighting the important role it has in osteoclast formation and function.^13^ More recently, it has also been shown that TRAF6 regulates the process of autophagy during RANKL‐induced osteoclast differentiation via the ubiquitination of Beclin 1.^14^ Autophagy is a highly conserved and important self‐catabolic cellular process. It can degrade damaged organelles and long‐lived proteins via the lysosomal system so that the raw materials could be recycled for reuse by the cell.^15^ Autophagy is induced during osteoclast formation in a RANKL‐dependent manner and is also important for osteoclast bone resorptive process.^16^ Therefore, pharmacological agents that can possess anti‐inflammatory properties and the ability to suppress the RANKL/RANK/TRAF6 signalling axis have great potential in the treatment and prevention of wear particle–induced osteolysis and other osteoclast‐mediated bone lytic diseases.

Nepetin, also known as Eupafolin or 6‐Methoxyluteolin, is a natural active flavonoid that existed in various plants containing the common sage herb.^17^ Nepetin has been proved to exhibit multiple biological effects possessing anti‐apoptotic, anti‐inflammatory, anti‐oxidant and anti‐cancer properties.^18^, ^19^ However, the effect of Nepetin on RANKL‐induced osteoclast formation and function has yet to be reported. Given the profound anti‐inflammatory and anti‐proliferative effect of Nepetin, we hypothesized that Nepetin might be a new pharmacological agent for the attenuation of inflammation‐induced osteoclast‐mediated osteolysis. In this research, we showed that Nepetin potently inhibited osteoclast differentiation and bone resorption in vitro and protected mice against Ti particle–induced calvarial osteolysis in vivo. Biochemical analyses found that Nepetin attenuated RANKL‐induced activation of the NF‐κB and MAPK signalling pathways, as well as TRAF6‐mediated ubiquitination of Beclin 1 which is necessary for the induction of autophagy. Thus, our study provided evidence for the potential therapeutic application of Nepetin against osteoclast‐mediated osteolytic diseases.

## 2 MATERIALS AND METHODS

### 2.1 Reagents and media

Nepetin (also known as Eupafolin or 6‐Methoxyluteolin) was purchased from ChromaDex (Los Angeles, CA, USA) and dissolved in dimethyl sulphoxide (DMSO). Foetal bovine serum (FBS) and alpha‐minimum essential medium (α‐MEM) were obtained from Gibco (Thermo Fisher Scientific, Waltham, MA, USA). Receptor activator of nuclear factor‐κB ligand (RANKL) and mouse recombinant macrophage‐colony‐stimulating factor (M‐CSF) were procured from R&D Systems (Minneapolis, MN, USA). Commercial grade titanium (Ti) particles in powdered form were from Alfa Aesar (Haverhill, MA, USA). Specific primary antibodies were obtained from Cell Signaling Technology (Danvers, MA, USA) or Abcam (Cambridge, UK). The antibodies used included IKKα, IKKβ, phosphorylated (p)‐IKKα/β, p‐IκBα, p‐p65, p‐ERK, p65, p‐JNK, p‐p38, IκBα, ERK, JNK, p38, NFATc1, c‐Fos, TRAF6, Beclin 1, LC3‐I/II, K63‐linkage specific polyubiquitin, TRAF3 and β‐actin.

### 2.2 Cell culture and in vitro osteoclast formation assay

Cells were removed from the femur and tibia of C57BL/6 mice (6‐week‐old), and cultured in complete α‐MEM (α‐MEM including 10% FBS) with M‐CSF (30 ng/mL) for 4 days or until 90% confluence. Adherent cells were considered to be bone marrow monocytes/macrophages (BMMs). The murine monocyte/macrophage cell line, RAW264.7 was obtained from the Chinese Academy of Sciences (Shanghai, China). For the dose‐dependent effect of Nepetin on osteoclast formation, BMMs cultured with complete α‐MEM with M‐CSF (30 ng/mL) were stimulated with RANKL (50 ng/mL) without or with 1.56, 3.125 or 6.25 μmol/L of Nepetin for 5 days. Culture media including RANKL, M‐CSF and Nepetin were changed every 2 days. For the time‐dependent effect of Nepetin on osteoclast formation, BMMs cultured with complete α‐MEM including M‐CSF (30 ng/mL) were stimulated with RANKL (50 ng/mL) and then treated with 6.25 μmol/L Nepetin on days 0–2, days 1–3, days 2–4 or days 3–5. DMSO (1:6000) was used as control. After the above treatment, the TRAP activity kit (Sigma‐Aldrich, St. Louis, MO, USA) was used to stain the cells using inverted optical microscope for image acquisition. ImageJ software (NIH, Bethesda, MD, USA) was used to quantify the number and area (percentage of a well) of TRAP‐positive osteoclasts (≥3 nuclei).

### 2.3 Cell viability assay

The effect of Nepetin on cell viability/proliferation was tested using the CCK‐8 assay kit (Beyotime Institute of Biotechnology, Shanghai, China). In brief, BMMs cultured with complete α‐MEM including M‐CSF (30 ng/mL) were stimulated with different concentrations (from 0.78 μmol/L to 200 μmol/L) of Nepetin for 48, 72, 96 or 120 hours. Then, CCK‐8 reagent (10 μL) was used to the culture dish and incubated for 3 hours. Then, the optical density was tested at 450 nm using the Multiskan FC Microplate Photometer (Thermo Fisher Scientific, Waltham, MA, USA).

### 2.4 Bone resorption assay

Sterilized and dried bovine bone discs were soaked in serum‐free α‐MEM at 4°C for 2 days and transferred to a 96‐well plate. BMMs were seeded onto collagen‐coated plates and cultured with complete α‐MEM with M‐CSF (30 ng/mL) and RANKL (50 ng/mL) for 3 days to form small pre‐osteoclastic cells. Cells were then removed from the collagen‐coated surface and centrifuged briefly for 5 minutes at 1000 × *g*, and then the same number of pre‐osteoclastic cells was seeded onto pre‐prepared bone discs and cultured with complete α‐MEM with RANKL and M‐CSF. Cells were allowed to attach for 6 hours and then treated without or with 1.56, 3.125 or 6.25 μmol/L Nepetin for 3 days. After the above treatment, the bone discs were air‐dried and gold‐plated for analysis under a Hitachi S‐4800 Field Emission Scanning Electron Microscope (Tokyo, Japan).

### 2.5 Quantitative real‐time polymerase chain reaction (qRT‐PCR)

Bone marrow monocytes/macrophages were cultured with complete α‐MEM containing M‐CSF (30 ng/mL) and RANKL (50 ng/mL) without or with 1.56, 3.125 or 6.25 μmol/L Nepetin. After 5 days of culture, total RNA was obtained using TRIzol reagent (Invitrogen, Carlsbad, CA, USA). RNA purity and concentration were tested by the NanoDrop One Microvolume UV‐Vis Spectrophotometer (Thermo Scientific). The RevertAid First Strand cDNA Synthesis Kit (Thermo Fisher Scientific) was used to carry on the progress of RNA reverse transcription. qRT‐PCR was carried out on the ABI 7500 Real‐Time PCR Detection System (Applied Biosystems, Foster City, CA, USA) with reaction mixtures containing cDNA template, SYBR Green qPCR Master Mix (Takara Bio, Shiga, Japan) and specific primer sets as follows: *c‐Fos* (Forward: 5′‐CCAGTCAAGAGCATCAGCAA‐3′; Reverse: 5′‐ AAGTAGTGCAGCCCGGAGTA‐3′); *NFATc1* (Forward: 5′‐ CCGTTGCTTCCAGAAAATAACA‐3′; Reverse: 5′‐ TGTGGGATGTGAACTCGGAA ‐3′); *calcitonin receptor* (*CTR*) (Forward: 5′‐TGCAGACAACTCTTGGTTGG‐3′; Reverse: 5′‐TCGGTTTCTTCTCCTCTGGA‐3′); *TRAP* (*ACP5*) (Forward:5′‐CACTCCCACCCTGAGATTTGT‐3′; Reverse: 5′‐AAGTAGTGCAGCCCGGAGTA‐3′); *V‐ATPase d2* (Forward: 5′‐AAGCCTTTGTTTGACGCTGT‐3′; Reverse: 5′‐TTCGATGCCTCTGTGAGATG‐3′); *DC‐STAMP* (Forward: 5′‐AAAACCCTTGGGCTGTTCTT‐3′; Reverse: 5′‐AATCATGGACGACTCCTTGG‐3′); *Beclin 1* (Forward: 5′‐ATGGAGGGGTCTAAGGCGTC‐3′; Reverse: 5′‐TGGGCTGTGGTAAGTAATGGA‐3′); *LC3* (Forward: 5′‐ GATAATCAGACGGCGCTT‐3′; Reverse: 5′‐ ACTTCGGAGATGGGAGTG ‐3′); *Atg5* (Forward: 5′‐TGTGCTTCGAGATGTGTGGTT‐3′; Reverse:5′‐ ACCAACGTCAAATAGCTGACTC‐3′); *Atg12* (Forward:5′‐ TGAATCAGTCCTTTGCCCCT‐3′; Reverse: 5′‐CATGCCTGGGATTTGCAGT‐3′); and *GAPDH* (Forward: 5′‐ACCCAGAAGACTGTGGATGG‐3′; Reverse: 5′‐CACATTGGGGGTAGGAACAC‐3′). PCR cycling conditions were set as 40 cycles of 95°C for 10 s, 60°C for 20 s and 72°C for 20 s. As previously described,^20^ the 2^−ΔΔCt^ method was used to measure gene expressions.

### 2.6 Western blot analyses

For the time‐dependent effect of Nepetin on early RANKL‐related signalling events, BMMs were pre‐treated without or with 6.25 μmol/L of Nepetin for 2 hours followed by treatment with RANKL (50 ng/mL）for 0, 5, 10, 20, 30 or 60 mins. For late RANKL‐induced signalling events, BMMs were cultured with complete α‐MEM including M‐CSF (30 ng/mL) and RANKL (50 ng/mL) without or with Nepetin (6.25 μmol/L) for 1, 3 or 5 days. For the dose‐dependent effect of Nepetin on RANLK‐related signalling events, BMMs were cultured with complete α‐MEM including M‐CSF (30 ng/mL) and RANKL (50 ng/mL) without or with Nepetin (1.56, 3.125 or 6.25 μmol/L) for 3 days. For the effects of Nepetin on autophagy, BMMs were pre‐treated with or without Nepetin (6.25 μmol/L) or rapamycin (200 nmol/L) for 2 hours followed by treatment with RANKL (50 ng/mL) for 30 minutes. After the treatment process above, cells were lysed by RIPA lysis buffer (Beyotime Institute of Biotechnology) containing a protease inhibitor, phenylmethanesulfonyl fluoride (PMSF). Cell lysates were collected, cleared by centrifugation and the supernatant containing post‐nuclear extracts were subjected to protein concentration quantification using the BCA assay (Beyotime Institute of Biotechnology). The protein samples were mixed with SDS‐PAGE loading buffer, denatured by boiling at 100°C, and protein samples were resolved in 10% SDS‐PAGE gel. Separated proteins were transferred onto the nitrocellulose membrane. Membranes were blocked with a QuickBlock blocking solution (Beyotime Institute of Biotechnology) for 10 minutes and then incubated with specific primary antibodies (diluted 1:1000) at 4°C overnight. Then membranes were treated with appropriate HRP‐conjugated secondary antibodies (diluted 1:10 000; Beyotime Institute of Biotechnology) for 1 hour. Protein‐antibody complexes were detected following exposure to Luminata Enhanced Chemiluminescence Reagent (Millipore Sigma, Billerica, MA, USA) and imaged on an ImageQuant LAS‐500 Imaging System (GE Life Sciences, Chicago, IL, USA). Densitometry of protein bands was measured using ImageJ software.

### 2.7 Immunofluorescence staining

BMM‐derived osteoclasts seeded on glass coverslips were cultured and stimulated with Nepetin as described in Western blot above. After the pre‐treatment process above, cells were kept in 4% PFA for 15 minutes, permeabilized in 0.5% Triton X‐100 for 30 minutes and then blocked with 5% appropriate normal animal serum for 30 minutes. After extensive washes, the primary antibodies (p65, LC3, TRAF6 or Beclin 1; diluted 1:100) were used to incubate with the fixed cells at 4°C overnight. For actin staining, cells were stained with phalloidin at 4°C for 1 hour and then proceeded with DAPI staining. The day after, cells were incubated with an appropriate fluorescent‐labelled secondary antibody in a dark place for 1 hour. Cells were stained with DAPI in the dark for 5 minutes. Coverslips were mounted, and fluorescence images were captured under by fluorescence or a confocal.

### 2.8 NF‐κB reporter gene assay

RAW264.7 cells were transfected with NF‐κB luciferase reporter plasmid (Genomeditech, Shanghai, China) using Lipofectamine™ 2000(lip 2000; Invitrogen). Cells were transfected and then pre‐treated with different concentrations of Nepetin (1.56, 3.125 or 6.25 μmol/L) for 2 hours and then stimulated with RANKL (50 ng/mL). After 6 hours of RANKL stimulation, cells lysed and luciferase activity in cellular extracts were measured on a luminometer using the Pierce Firefly Luciferase Assay System (Thermo Scientific).

### 2.9 Co‐immunoprecipitation (Co‐IP)

BMMs were pre‐treated without or with Nepetin (6.25 μmol/L) for 2 hours followed by treatment with RANKL (50 ng/mL) for 30 minutes. After RANKL stimulation, cells were lysed by Western and IP lysis buffer supplemented with PMSF protease inhibitor for 30 minutes. Cell lysates were cleared by centrifugation, and 500 μg of total protein was treated with anti‐TRAF6 (5 μg) or anti‐Beclin 1 (5 μg) antibodies overnight at 4°C under constant and gentle rotation. Total protein incubated with non‐specific IgG antibody was applied as a negative control. Subsequently, 20 μL of protein A/G‐sepharose beads (Cell Signaling Technology) was added to each sample and then incubated at 4°C overnight under constant and gentle rotation. The beads were denatured by boiling in 2× SDS loading buffer for 5 minutes. After centrifugation, proteins were resolved on 10% SDS‐PAGE gels.

### 2.10 Titanium particle–induced calvarial osteolysis

Twenty‐four C57BL/6 male mice (6‐week‐old) were obtained from the Laboratory Animal Center of Zhejiang University (Hangzhou, Zhejiang, China). The Animal Experimental Ethics Committee of the Taizhou Hospital of Zhejiang Province (Zhejiang, China) gave its approval to all experimental schemes. Mice were randomly divided into 4 groups (6 in each group): negative control sham group (PBS treatment), positive control Ti‐particle implantation group (vehicle; PBS treatment; vehicle), Ti‐particle + low‐dose Nepetin group (0.5 mg/kg) and Ti‐particle + high‐dose Nepetin group (1.0 mg/kg). Following anaesthesia with intraperitoneal injections of 1% pentobarbital sodium (50 mg/kg), a 1‐cm sagittal incision was made in the middle of the skull with a sharp blade under sterile conditions. Thirty micrograms of Ti particles were embedded under the periosteum and onto the surface around the midline suture of the calvarial bone, and then the skin was sutured closed. The sham control group received surgical operation but no implantation of Ti particles. Two days after Ti particle implantation, Nepetin at 0.5 or 1 mg/kg was intraperitoneally injected every other day for 14 days. Meanwhile, mice in the sham control and vehicle groups were injected with an equal amount of PBS every other day. There were no adverse effects or death throughout the experimental period. After 14 days, all calvaria of experimental mice were surgically removed and kept in a 4% PFA before processed for micro‐CT (computed tomography) and histological assessments.

### 2.11 Micro‐CT scanning

All calvarial bone samples were scanned by the Scanco Medical micro‐CT 100 high‐resolution scanners (Brüttisellen, Switzerland). The parameters of scanning and image acquisition are as follows: 70 kV voltage, 200 μA current, 20 μm isometric resolution. After three‐dimensional reconstruction with associated software, a square region where Ti particles were implanted was chosen for further analysis.

### 2.12 Histology and immunohistochemistry

Following micro‐CT analyses, fixed calvarial bone tissues were decalcified in 10% EDTA, dehydrated in sequential immersion in 75%, 85%, 95% and 100% ethanol solutions, cleared in xylene and embedded in paraffin blocks. The sections were manufactured on a Leica SM2010 R Sliding (Sledge) Microtome (Leica Biosystems, Wetzlar, Germany) and used to haematoxylin and eosin (H&E) and TRAP staining according to standard laboratory protocols. Briefly for H&E staining, tissue sections were deparaffinized and immersed in the H&E dye for 3 minutes. Tissue sections were then dehydrated in isopropyl alcohol and mounted. For TRAP staining, tissue sections must be dewaxed with xylene, rehydrated by immersion in sequential graded alcohol (100%, 95%, 85% and 75%) and then soaked in 3% hydrogen peroxide/methanol solution for 15 minutes. Tissue sections were then immersed in the TRAP staining solution and incubated for 60 minutes in a dark place. All tissue sections were observed under an optical light microscope.

For immunohistochemical staining, dewaxed and rehydrated tissue sections were submitted to heat‐induced antigen retrieval. Tissue sections were soaked in 0.5% Triton X‐100 for 20 minutes, transferred to a wet box and soaked in 3% hydrogen peroxide/methanol solution for 30 minutes. The sections were blocked using 10% normal goat serum for 30 minutes before treated with antibody (p65 or LC3; diluted 1:400) overnight at 4°C. Tissue sections were treated with an appropriate secondary antibody for 1 h. Staining was developed by incubation with 3,5‐diaminobenzidine (DAB) substrate for 10 mins, counterstained with haematoxylin for 3 minutes, differentiated with 1% hydrochloric acid, dehydrated by gradient alcohol, vitrification by dimethyl benzene, and finally sealed. The optical microscope was used for observation of sections.

### 2.13 Statistical analysis

All experimental data are shown as mean ± SEM. The independent sample Student's *t* test was applied for comparison between two groups. One‐way analysis of variance (ANOVA) was applied for comparison between multiple groups with LSD and SNK post hoc tests. The GraphPad Prism 8.0 (San Diego, CA, USA) was applied for statistical analyses. *P*‐values <0.05 or unless otherwise specified were considered statistically significant.

## 3 RESULTS

### 3.1 Nepetin inhibits osteoclast differentiation in vitro

**FIGURE 1:**
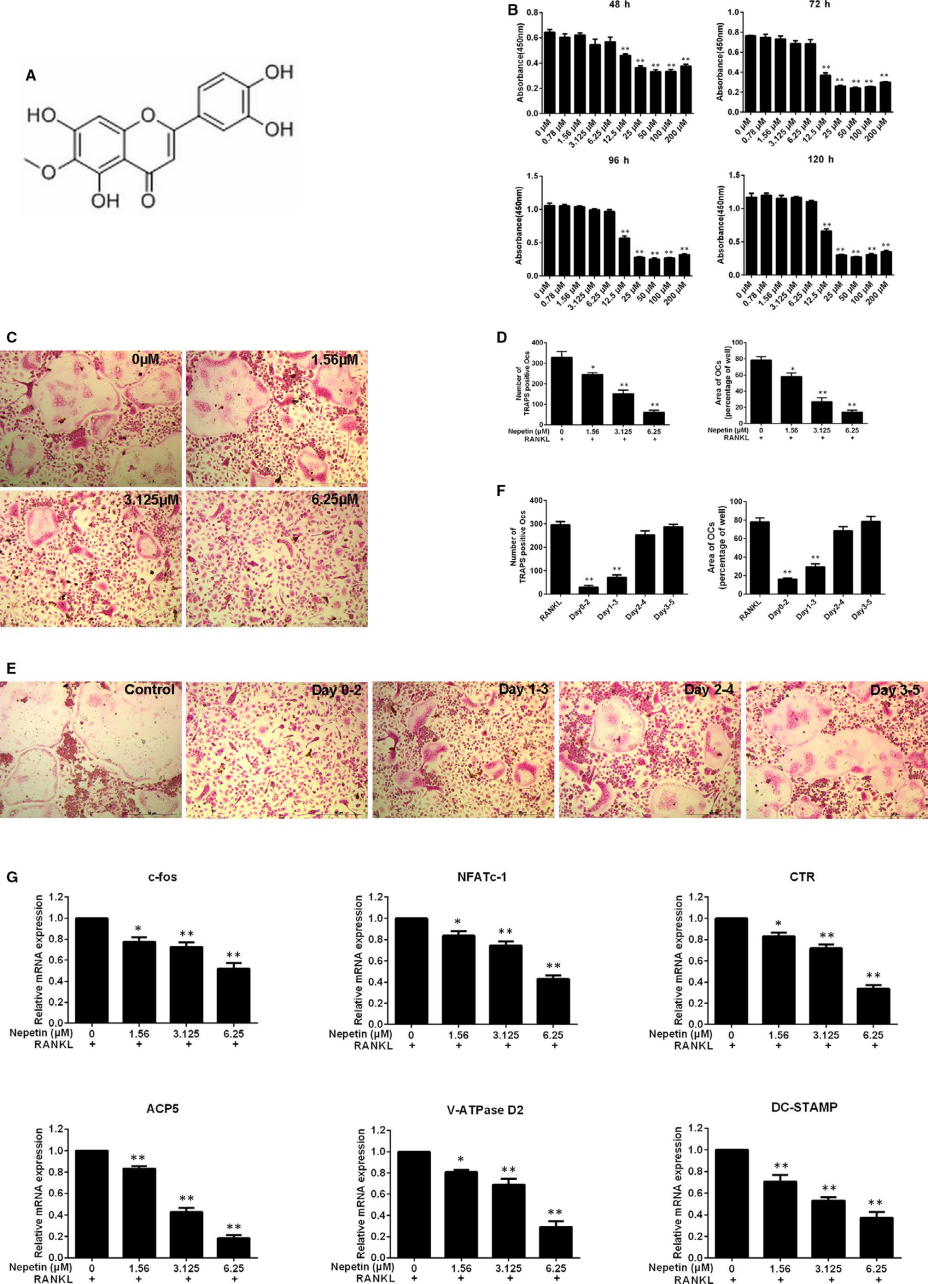
Nepetin inhibits RANKL‐induced osteoclastogenesis in vitro. (A) Structure of Nepetin. The molecular formula of C16H12O7 and an average mass of 316.26228 Da. (B) BMMs were cultured with different concentrations of Nepetin for 48, 72, 96 and 120 h. The viability by CCK‐8 assay was evaluated. (C) BMMs were stimulated without or with Nepetin (1.56, 3.125 and 6.25 μmol/L) in osteoclastic differentiation medium, and then processed with TRAP staining on day 5. (D) The area and number of TRAP‐positive cells (nuclei ≥ 3) were quantified. (E) BMMs were cultured in complete α‐MEM including M‐CSF (30 ng/mL) and RANKL (50 ng/mL) and then treated with 6.25 μmol/L Nepetin on days 0‐2, 1‐3, 2‐4 or 3‐5. (F) The area and number of TRAP‐positive cells were quantified. (G) BMMs were cultured with RANKL without or with 1.56, 3.125 or 6.25 μmol/L Nepetin for 5 d. The qRT‐PCR was used to evaluate the effect of Nepetin on c‐fos, NFATc1, ACP5, CTR, DC‐STAMP and V‐ATPase D2 mRNA expression. *P < 0.05; **P < 0.01

We first found the cellular cytotoxicity of Nepetin (molecular structure shown in Figure 1A) against BMM cells using the CCK‐8 assay. BMMs were conditioned with various concentrations of Nepetin for 48, 72, 96 and 120 hours. Nepetin at concentrations of 6.25 μmol/L and below did not affect the activity of BMMs at all time‐points tested indicating low to no cellular cytotoxicity at these concentrations (Figure 1B). On the other hand, concentrations of 12.5 μmol/L and above significantly inhibited the viability of BMMs (Figure 1B). Based on these results, the concentration of Nepetin selected for use in subsequent cellular and biochemical in vitro examinations was 1.56, 3.125 and 6.25 μmol/L.

Next, we examined the dose‐dependent effect of Nepetin on RANKL‐induced osteoclast formation. BMMs were induced to form multinucleated osteoclasts in the presence of RANKL without or with indicated concentrations of Nepetin for 5 days. As shown in Figure 1C,D, Nepetin treatment dose‐dependently impaired the number and size of TRAP‐positive osteoclast formed. To define the stage of osteoclast formation to which Nepetin exerts its inhibitory action, M‐CSF‐dependent BMMs were cultured with RANKL and then treated with 6.25 μmol/L Nepetin on the appointed days. As shown in Figure 1E,F, treatment with Nepetin during the early stages of osteoclast differentiation, that is between days 0 and 3, resulted in significant inhibition of osteoclast formation. Conversely, treating BMMs with Nepetin at the later stages between days 3 and 5 did not markedly affect RANKL‐induced osteoclast formation. Analysis of osteoclast marker gene expression at day 5 of RANKL stimulation confirmed the anti‐osteoclastogenic effect of Nepetin (Figure 1G). The gene expression of *c‐Fos*, *NFATc1*, *CTR*, *ACP5* (TRAP), *ATP6V0d2* (V‐ATPase D2) and *DC‐STAMP* was dose‐dependently down‐regulated by Nepetin treatment. Collectively these results show that the sublethal concentration of piperidine not only effectively inhibits the formation of osteoclasts induced by RANKL, but also inhibits the expression of osteoclast‐related marker genes.

### 3.2 Nepetin suppresses precursor cell fusion and impairs mature osteoclast bone resorption in vitro

**FIGURE 2:**
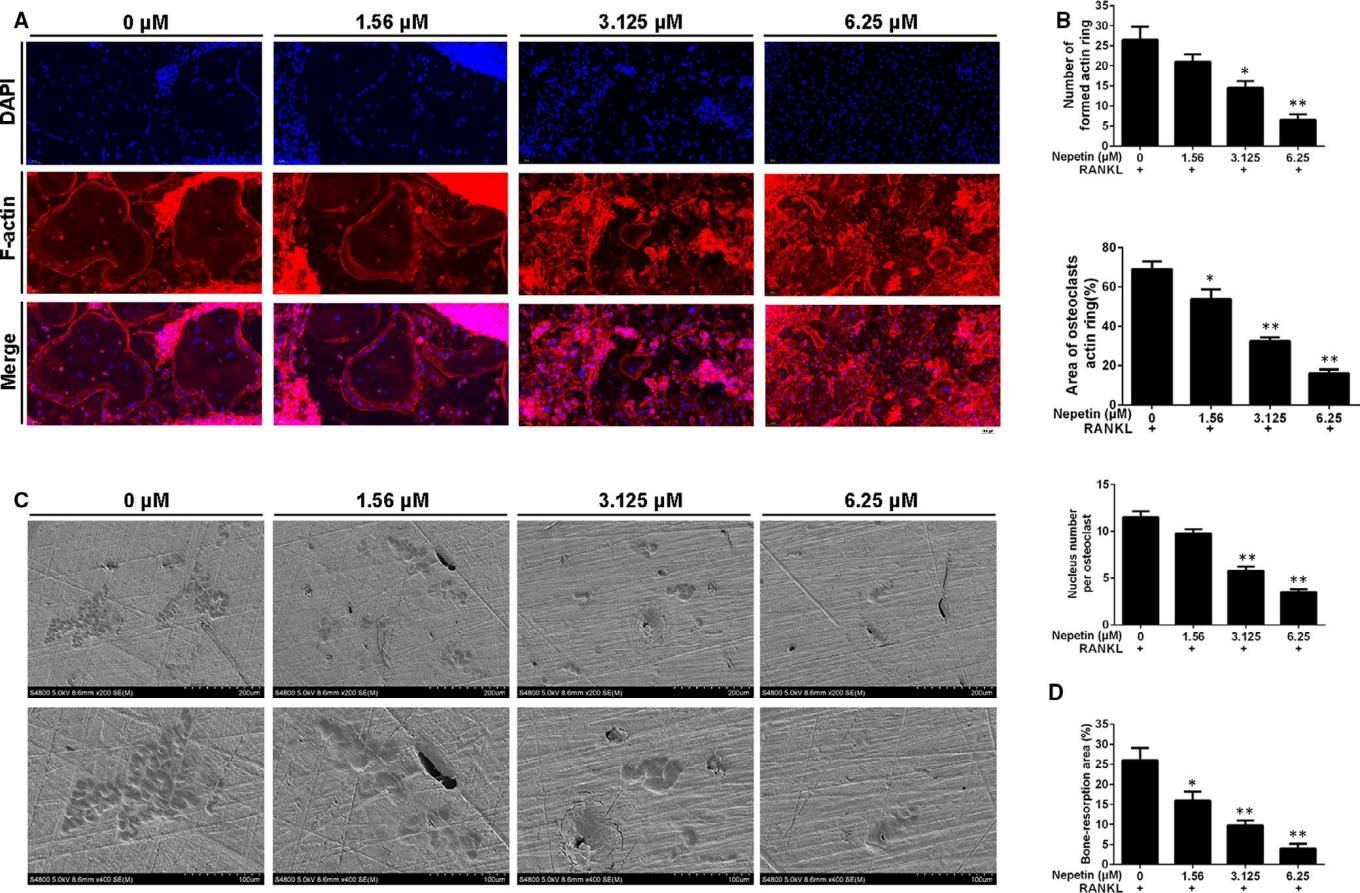
Nepetin suppresses the fusion of precursor cells and impairs the bone resorption of mature osteoclast. (A) BMMs cultured with complete α‐MEM including M‐CSF (30 ng/mL) and RANKL (50 ng/mL) without or with Nepetin (1.56, 3.125 or 6.25 μmol/L) for 5 d were stained for laser scanning confocal microscopy observation. (B) Quantification of the area of osteoclasts actin ring (%) and the number of formed actin rings. (C) The pre‐osteoclastic cells seeded onto bone discs in complete α‐MEM containing RANKL (50 ng/mL) and M‐CSF (30 ng/mL) were cultured without or with Nepetin (1.56, 3.125 or 6.25 μmol/L) for 3 d. (D) Quantification by ImageJ of the percentage of bone resorption area. *P < 0.05; **P < 0.01

The down‐regulation of the expression of *DC‐STAMP* and *ATP6V0d2* gene suggests that Nepetin suppresses BMM precursor fusion, a prerequisite step in the multinucleation of mature osteoclasts. Hence, we employed immunofluorescence analysis to further examine the cytoskeletal morphology and nucleation of osteoclasts in the presence of Nepetin. Osteoclasts were fixed and stained with phalloidin for actin cytoskeleton (red) and DAPI nuclei stain (blue). As shown in Figure 2A, untreated osteoclasts exhibit a distinct podosome actin belt that circumscribes the well‐spread mature osteoclasts. On the other hand, osteoclasts derived from BMMs treated with increasing concentrations of Nepetin showed a dose‐dependent decrease in the number and size of the podosome actin belt (Figure 2A,B). Quantification of the number of nuclei per osteoclasts in the Nepetin‐treated cells further showed a reduction in multinucleation with many mononuclear cells in the 6.25 μmol/L treatment group (Figure 2A,B). These results further suggest that Nepetin inhibited osteoclast formation by impairing BMM precursor cell fusion.

The formation of the podosome actin belt is important in the subsequent formation of the F‐actin ring in polarized osteoclasts, that is, osteoclasts that are activated towards bone resorption. Thus, we next assessed whether Nepetin treatment affected the ability of mature polarized osteoclasts to resorb bone. BMM‐derived osteoclasts cultured on bovine bone discs were treated with indicated concentrations of Nepetin for 3 days. As shown in the scanning electron micrographs of bone resorption in Figure 2C,A marked reduction in the ability of Nepetin‐treated osteoclast to resorb bone was observed. Quantitative measurement of the percentage of resorption area relative to total bone disc area further revealed a dose‐dependent decrease in osteoclast bone resorption following treatment with Nepetin (Figure 2D). Collectively, these results indicate that Nepetin also exerts anti‐resorptive effects on mature osteoclast bone resorption in vitro.

### 3.3 Nepetin impairs RANKL‐induced early activation of the NF‐κB signalling pathway and attenuates the induction of c‐Fos and NFATc1

**FIGURE 3:**
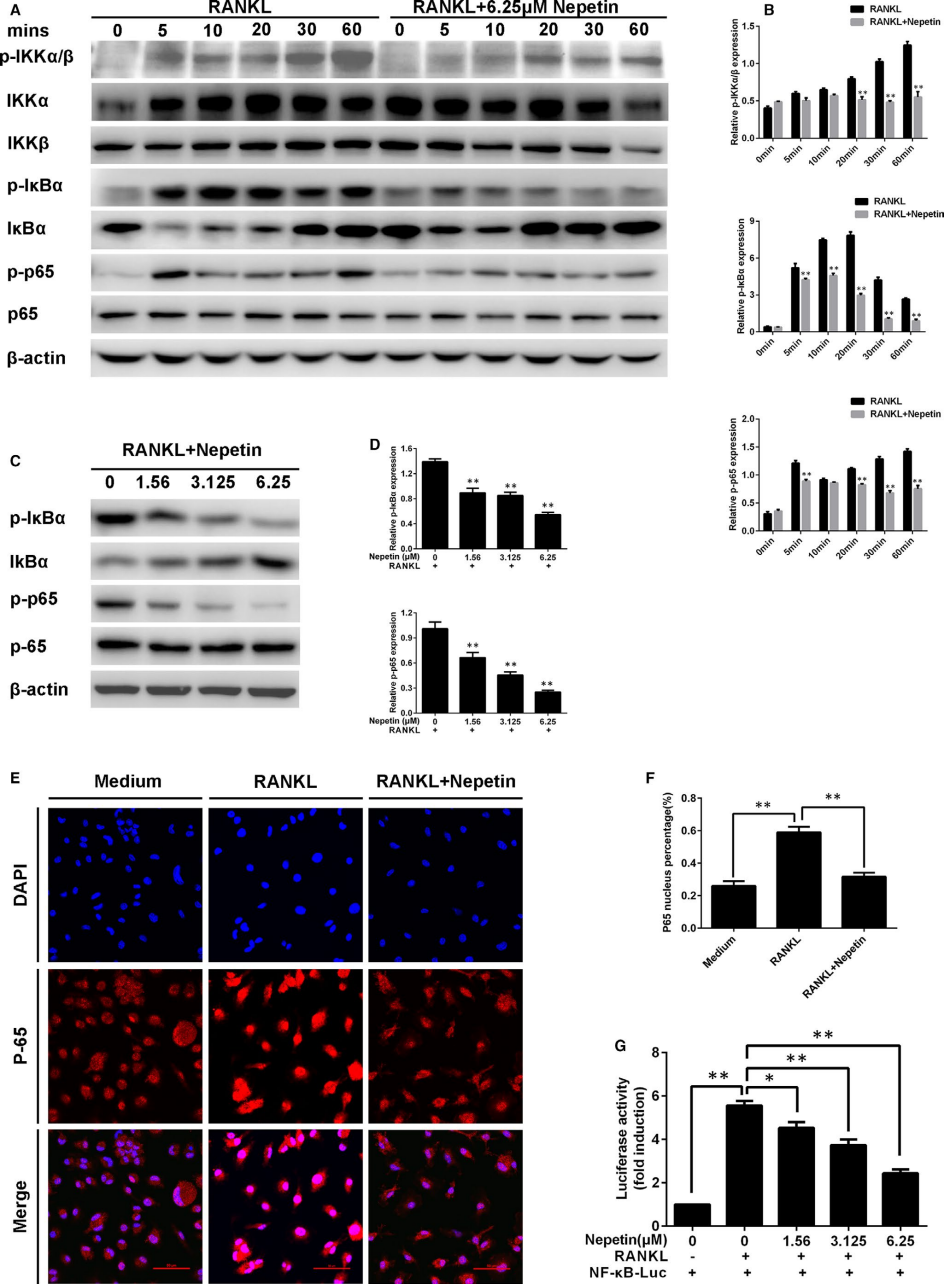
Nepetin impairs RANKL‐induced activation of the NF‐κB signalling pathway. (A) BMMs were pre‐treated without or with 6.25 μmol/L of Nepetin for 2 h followed by treatment with RANKL (50 ng/mL) for 0, 5, 10, 20, 30 or 60 min. Representative immunoblot obtained with IKKα, IκBα, IKKβ, p65, p‐IKKα/β, p‐IκBα, p‐p65 and β‐actin antibodies. (B) Densitometric analysis for (A) showing the relative expression of p‐IKKα/β, p‐IκBα and p‐p65. (C) BMMs were cultured with complete α‐MEM including M‐CSF (30 ng/mL) and RANKL (50 ng/mL) without or with 1.56, 3.125 or 6.25 μmol/L Nepetin for 3 d. (D) Densitometric analysis for (C) showing the relative expression of p‐p65 and p‐IκBα.(E) BMMs were pre‐treated without or with Nepetin (6.25 μmol/L) for 2 h, and cultured with RANKL (50 ng/mL) for 30 min. The cells were stained for laser scanning confocal microscopy assay. (F) The ratio of the nucleus fluorescence intensity to whole‐cell fluorescence intensity. (G) RAW264.7 cells were transfected with NF‐κB luciferase reporter plasmid, pre‐treated with or without Nepetin (1.56, 3.125 or 6.25 μmol/L) for 2 h, and then cultured with RANKL (50 ng/mL) for 6 h. *P < 0.05; **P < 0.01

Osteoclast formation in response to RANKL requires the activation of various downstream signalling pathways that culminates in the induction of transcriptional factors c‐Fos and NFATc1. Of the earliest RANKL‐induced signalling events activated, the NF‐κB and MAPK are two of the principal pathways that have been extensively studied. Using Western blot analyses, we tested the influence of Nepetin on the early stimulation of these two crucial signalling pathways. RANKL stimulation induces the rapid activation of IKK complex, allowing it to phosphorylate inhibitory κB proteins which bind and sequesters p65/p50 NF‐κB subunits in the cytoplasm thereby inhibiting its transcriptional function. Phosphorylated IκB's are rapidly degraded via the proteasomal pathway releasing the p65/p50 NF‐κB subunits to translocate to the nucleus to activate gene transcription. As shown in Figure 3A, RANKL stimulation induces the rapid activation phosphorylation of IKKα within 5 minutes leading to the subsequent phosphorylation and degradation of IκBα within the same timeframe. At the same time, p65 is also phosphorylated which then allows it to translocate to the nucleus. On the other hand, Nepetin treatment significantly blocked the phosphorylation of IKKα, IκBα and p65 suggesting impaired activation of the NF‐κB signalling (Figure 3A,B). In a similar fashion, the activation of NF‐κB after 3 days of RANKL stimulation was similarly and dose‐dependently impaired following Nepetin treatment (Figure 3C,D). Using immunofluorescence analysis, we further showed that the nuclear translocation of p65 was markedly reduced in the presence of Nepetin (Figure 3E,F). This was further confirmed using luciferase gene reporter assay which demonstrated a dose‐dependent decrease in NF‐κB activity following Nepetin treatment (Figure 3G). Thus collectively, these results suggest that Nepetin inhibits RANKL‐induced activation of NF‐κB via the upstream inhibition of IKK activation and as a result prevents IκBα degradation and p65 activation and nuclear localization, which consequently impairs NF‐κB transcriptional activity.

**FIGURE 4:**
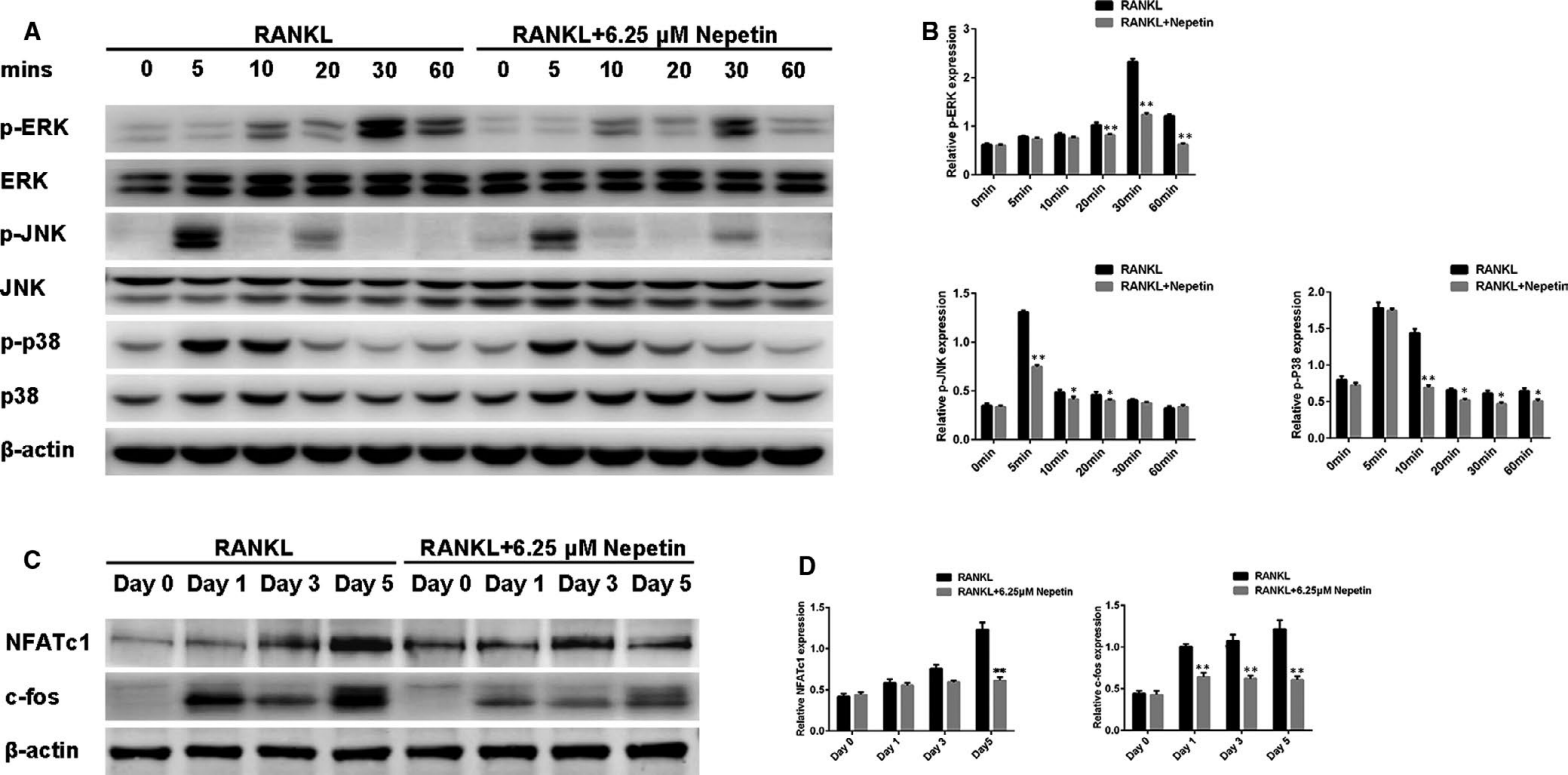
Nepetin impairs RANKL‐induced activation of the MAPK signalling pathway and attenuates the induction of NFATc1 and c‐fos. (A) BMMs were pre‐treated without or with Nepetin (6.25 μmol/L) for 2 h, and then cultured with RANKL (50 ng/mL) for 0, 5, 10, 20, 30 or 60 mins. Representative immunoblot obtained with p‐JNK, p‐ERK, p‐p38, JNK, ERK, p38 and β‐actin antibodies. (B) Densitometric analysis for (A) showing the relative expression of p‐p38, p‐JNK and p‐ERK. (C) BMMs with or without 6.25 μmol/L Nepetin were cultured with M‐CSF (30 ng/mL) and RANKL (50 ng/mL) for 1, 3 or 5 d. Representative immunoblot obtained with c‐fos, NFATc1 and β‐actin antibodies. (D) Densitometric analysis for (C) showing the relative expression of NFATc1 and c‐fos. *P < 0.05; **P < 0.01

As with the rapid activation of NF‐κB, RANKL stimulation similarly activates the MAPK signalling pathway including ERK, JNK and p38. However, unlike the potent inhibitory effect of Nepetin on NF‐κB activation, the activation of all three MAPK pathways was only moderately inhibited (Figure 4A,B). This suggests that the inhibitory effect of Nepetin is predominantly via the impairment of the NF‐κB pathway. The timely and efficient activation of NF‐κB and MAPK pathways is important in the downstream induction of c‐Fos and NFATc1 (Figure 4C, RANKL control), two transcription factors that are important for osteoclast differentiation. Particularly NFATc1 (the master transcription factor) controls the expression of many osteoclast genes including the genes examined in Figure 1G. Consistent with impaired NF‐κB activation, the subsequent downstream induction of both c‐Fos and NFATc1 was significantly reduced following Nepetin treatment (Figure 4C,D). Thus as a result of the impairment of NF‐κB activation following Nepetin treatment, the induced effect of c‐Fos and NFATc1 was consequently attenuated.

### 3.4 Nepetin inhibits autophagy activation via the TRAF6‐Beclin 1 pathway

**FIGURE 5:**
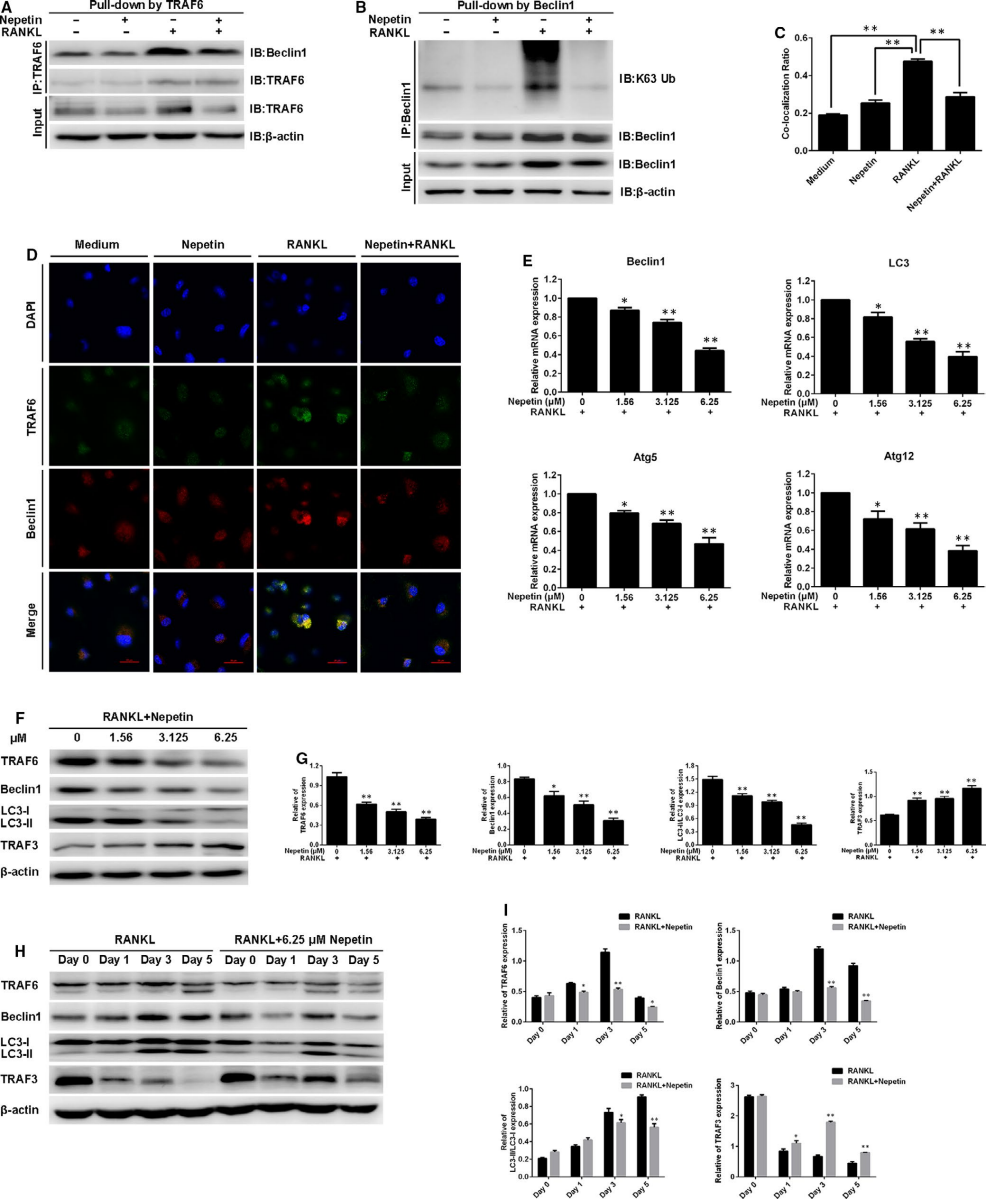
Nepetin obstructs RANKL‐induced autophagy by blocking TRAF6‐mediated ubiquitination of Beclin 1. (A) BMMs were pre‐incubated with Nepetin (6.25 μmol/L) for 2 h followed by treatment with RANKL (50 ng/mL) for 30 min. The effect of Nepetin on TRAF6‐Beclin 1 interaction was determined by Co‐IP. (B) The experiment was performed as described in A. The effect of Nepetin on ubiquitination of Beclin 1 was determined by Co‐IP. (C) The co‐localization ratio of TRAF6 and Beclin 1 was calculated by Pearson's correlation coefficient. (D) BMMs were treated as shown in A. BMMs were incubated with TRAF6 with anti‐TRAF6 antibody (rabbit origin) and anti‐Beclin 1 antibody (mouse origin) followed by incubation with Alexa Fluor 488 (green)–conjugated goat (anti‐rabbit) and Alexa Fluor 594 (red)–conjugated goat (anti‐mouse). The cells stained for laser scanning confocal microscopy observation. (E) BMMs cultured with RANKL (50 ng/mL) without or with Nepetin (1.56, 3.125 or 6.25 μmol/L) for 5 d. The qRT‐PCR was used to evaluate the effect of Nepetin on Beclin 1, LC3, Atg5 and ATg12 mRNA expression. (F) BMMs were cultured with RANKL (50 ng/mL) with or without Nepetin (1.56, 3.125 or 6.25 μmol/L) for 3 d. Representative immunoblot obtained with TRAF6, Beclin 1, LC3‐I/II, TRAF3 and β‐actin antibodies. (G) Densitometric analysis for (F) showing the expression of TRAF6, Beclin 1, LC3‐II/LC3‐I and TRAF3. (H) Experiments were performed as shown in Figure 4C. Representative immunoblot obtained with TRAF6, Beclin 1, LC3‐I/II, TRAF3 and β‐actin antibodies. (I) Densitometric analysis for (H) showing the expression of TRAF6, Beclin 1, LC3‐II/LC3‐I and TRAF3. *P < 0.05; **P < 0.01

**FIGURE 6:**
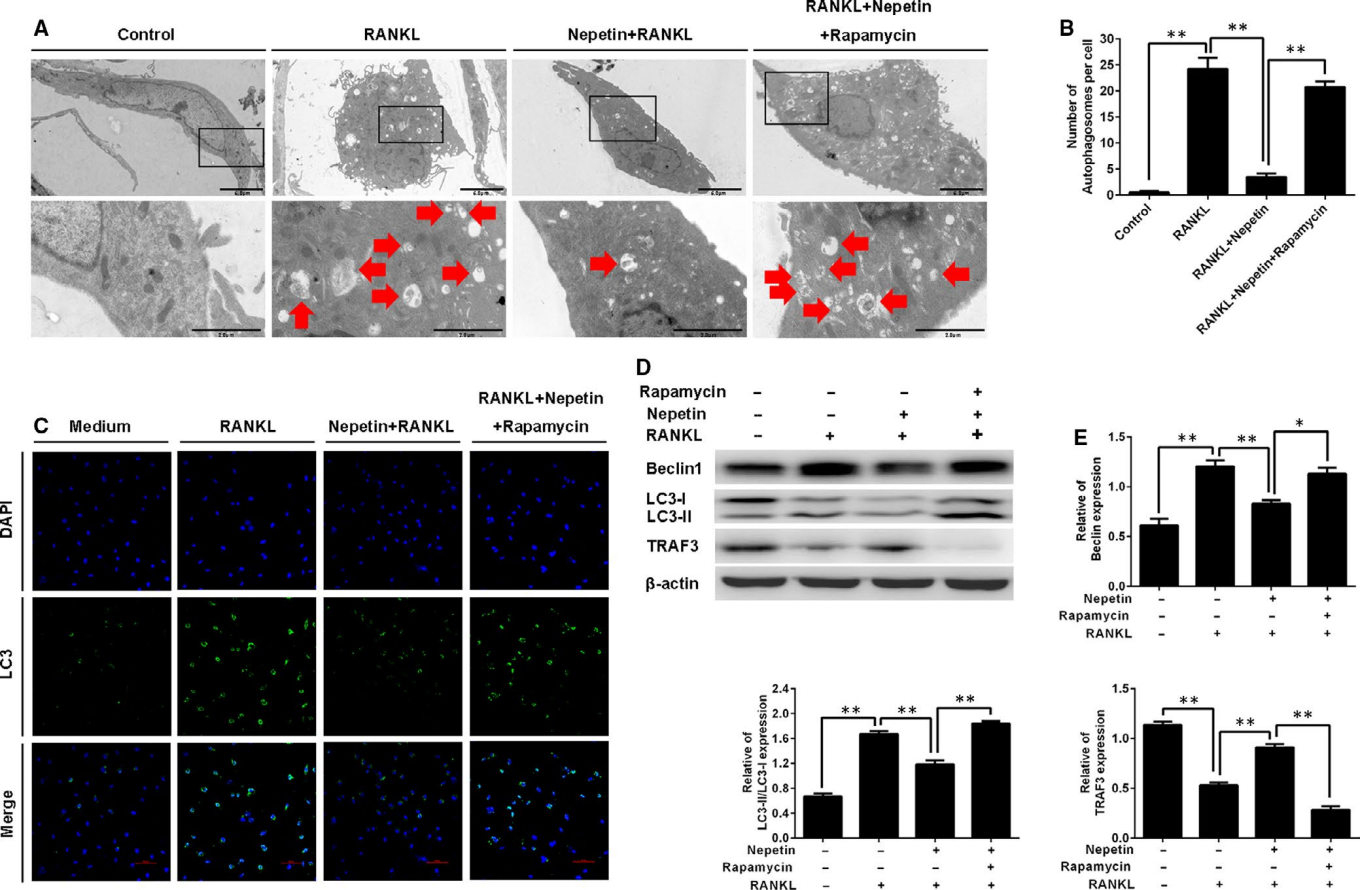
Nepetin inhibits RANKL‐mediated autophagic activation. (A) BMMs were pre‐incubated with rapamycin（200 nmol/L）for 2 h and then cultured with RANKL (50 ng/mL) and Nepetin（6.25 μmol/L）for 3 d. The TEM image of autophagosomes (Arrow). (B) The number of autophagosomes was quantified using ImageJ. (C) BMMs were stimulated with or without Nepetin (6.25 μmol/L) or rapamycin (200 nmol/L) 2 h followed by treatment with RANKL (50 ng/mL) for 30 min and then stained. The laser scanning confocal microscope for image observation. (D) Experiments were performed as shown in (C). Representative immunoblot obtained with Beclin 1, LC3‐I/II, TRAF3 and β‐actin antibodies. (E) Densitometric analysis for (D) showing the expression of Beclin 1, LC3‐I/II and TRAF3. *P < 0.05; **P < 0.01

Recent studies have found that the autophagic process is important for RANKL‐induced osteoclast differentiation and function,^21^ with TRAF6‐mediated ubiquitination of Beclin 1 crucial for the induction of autophagy and osteoclast differentiation.^14^ TRAF6 is a unique E3 ubiquitin ligase essential for the transduction of RANKL‐RANK signalling events.^22^ Using Co‐IP, we confirmed that TRAF6 interacts with Beclin 1 in a RANKL‐dependent manner and that binding of TRAF6 induces ubiquitination of Beclin 1 (Figure 5A,B). This is consistent with the finding by *Arai and colleagues (2019)*. Interestingly, the treatment of BMM cells with Nepetin diminished the TRAF6‐Beclin 1 interaction and completely abolished TRAF6‐mediated ubiquitination of Beclin 1 (Figure 5A,B). Inhibition of TRAF6‐Beclin 1 interaction by Nepetin was further verified using immunofluorescence co‐localization analysis. Compared with RANKL only treated BMMs, which shows intense co‐localization of TRAF6‐Beclin 1, Nepetin treatment completely prevented the co‐localization signal (Figure 5C,D). These data suggest that the induction of autophagy by RANKL is likely to be inhibited by Nepetin treatment. In line with this, the expression of autophagy‐related genes including *Beclin 1*, *LC3*, *Atg5* and *Atg12* was dose‐dependently down‐regulated (Figure 5E). A similar dose‐dependent (Figure 5F,G) and time‐dependent (Figure 5H,I) inhibition in the protein expression of TRAF6, Beclin 1 and LC3‐1/II was observed. On the other hand, the expression of TRAF3 which normally undergoes degradation via the autophagy system following RANKL stimulation was dose‐dependently elevated following Nepetin treatment, which further confirms the inhibition of autophagic process (Figure 5F‐I). Using transmission electron microscopy and immunofluorescence analyses, we further observed a significant reduction in autophagic vacuoles or autophagosomes (LC3‐conjugated) following Nepetin treatment (Figure 6A‐C). These results collectively suggest that Nepetin can obstruct RANKL‐induced autophagy by blocking TRAF6‐mediated ubiquitination of Beclin 1. Interestingly, however, the inhibitory effects of Nepetin on RANKL‐induced autophagy can be suppressed by co‐stimulation with rapamycin, a potent pharmacological inducer of autophagy (Figure 6A‐E). Rapamycin functions upstream of Beclin 1 via the inhibition of mTOR signalling, a pathway distinct from TRAF6, suggesting an alternative activation pathway for autophagy.

### 3.5 Nepetin protects against titanium (Ti) particle–induced calvarial osteolysis

**FIGURE 7:**
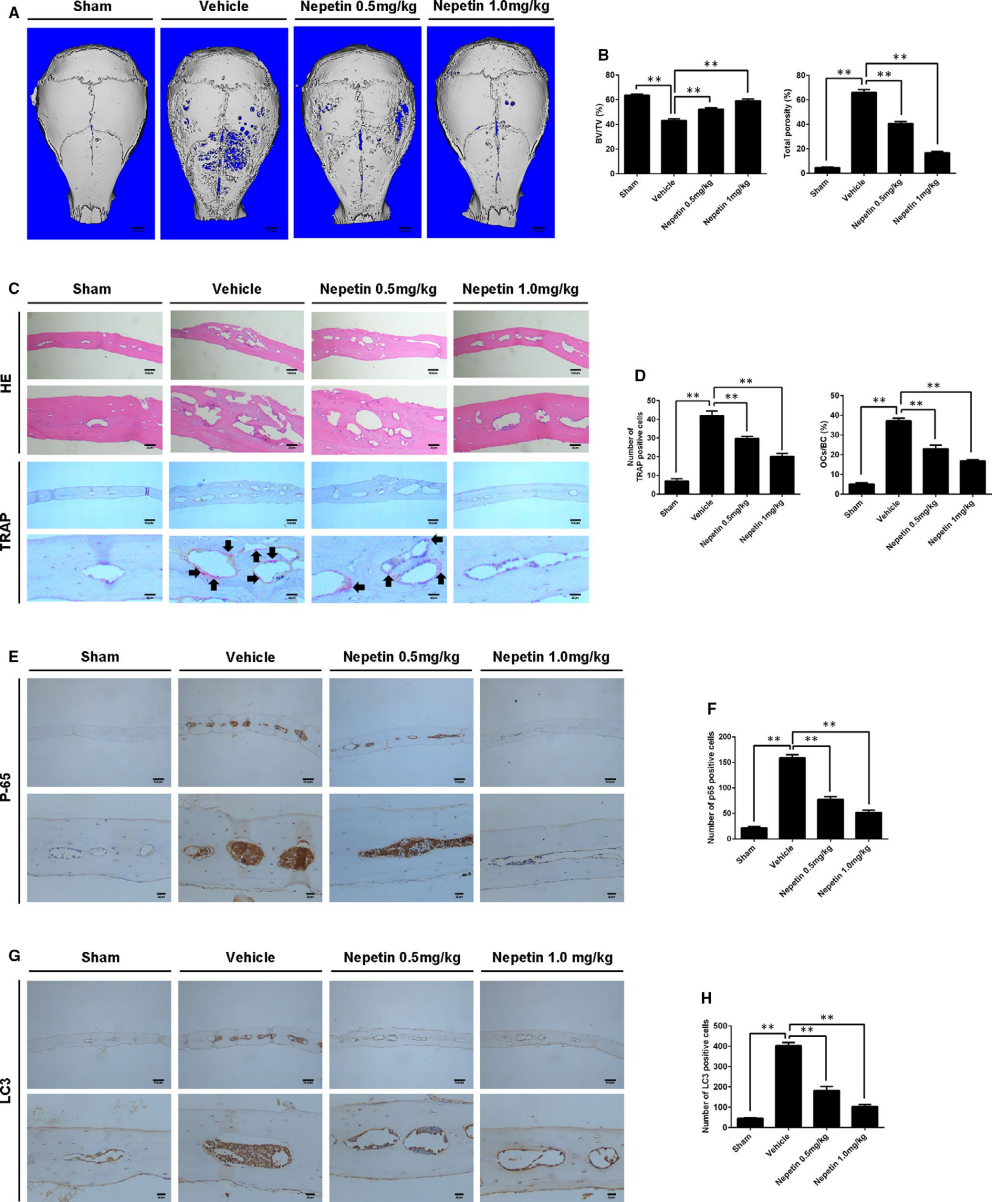
Nepetin protects against Ti particle–induced calvarial osteolysis. (A) The 3D reconstruction image of mice calvaria. (B) The total porosity and bone volume as a proportion of total tissue volume (BV/TV, %) from each group were measured. (C) The TRAP and H&E of calvarial slices. (D) The number of TRAP‐positive osteoclasts and the percentage of osteoclast surface per bone surface (OCs/BS, %) were quantified using ImageJ. (E) P65 immunohistochemistry of mice calvaria. (F) The number of p65 positive cells was quantified using ImageJ. (G) LC3 immunohistochemistry of mice calvaria. (H) The number of LC3‐positive cells was quantified using ImageJ. **P < 0.01

With these promising in vitro cellular and biochemical results, we next examined whether in vivo administration of Nepetin will exert any therapeutic benefits against osteoclast‐mediated pathological bone destruction using the mouse model of Ti particle–induced calvarial osteolysis. Compared with negative Sham controls, extensive bone destruction with aplenty of eroded voids around the midline suture where Ti particles were implanted were observed in the vehicle group (Ti‐particle injection) (Figure 7A). Abundant inflammatory cell infiltration was also observed (Figure 7B). Treatment with Nepetin dose‐dependently reduced Ti particle–induced osteolysis with potent protective effects observed at the high‐dose administration (Figure 7A,B; 3D reconstructions and HE staining, respectively). Morphometric analyses of bone parameters confirmed the significant improvement in bone volume (BV/TV, %) and reduction in the percentage of total porosity (Figure 7C). TRAP staining of the calvarial bone sections revealed abundant multinucleated osteoclasts at the site of Ti particle injection and lining the bone surface. On the other hand, the total number of osteoclasts lining the bone was dose‐dependently reduced following Nepetin administration, as well as TRAP‐positive osteoclasts (Figure 7B). This observation was further confirmed by histomorphometric analyses (Figure 7D). The reduction in osteoclast number in the Nepetin‐treated groups was found to be associated with decreased p65 expression (Figure 7E,F) and reduced autophagic response (decreased LC3 expression) (Figure 7G,H), which is consistent with our in vitro findings. Thus taken together, these provide promising evidence for the protective effects of Nepetin against excessive osteoclast‐mediated bone destruction and may serve as an effective anti‐osteoclastic and anti‐resorptive agent in the treatment of osteolytic bone conditions.

## 4 DISCUSSION

Aseptic prosthetic loosening leading to implant failure is a common complication of artificial joint arthroplasty.^23^ However, the cause of aseptic loosening is not fully understood but metal wear‐debris particles arising from the articulating surfaces at the implant‐bone interfaces have been shown to provoke the inflammatory response in the bone tissue near and around the prosthesis stimulates bone osteolysis and loosening of the implant.^24^ The molecular interplay governing inflammatory response and osteoclast‐mediated bone destruction is complex often involving the production of chemokines and cytokines, and cell‐cell interactions that enhance osteoclast recruitment, formation and activity adjacent to and surrounding the bone‐implant interface causing localized bone destruction.^25^ Current pharmacological interventions including bisphosphonates, oestrogen replacements and anti‐RANKL antibody (Denosumab) exhibit somewhat beneficial effects against osteoclast‐mediated osteolysis.^23^, ^26^ However, serious undesirable side effects including cardiovascular events, nephrotoxicity,^27^ osteonecrosis of the jaw,^28^ atypical fractures^29^ and malignant tumour formation are becoming increasingly prominent limiting their long‐term use. Therefore, the identification of safer more effective therapeutic agents is urgently demanded.

Nepetin, also known as Eupafolin or 6‐Methoxyluteolin, is a natural active flavonoid.^30^ It has been confirmed to possess potent anti‐inflammatory effects as well as anti‐oxidant and anti‐tumorigenic effects.^18^, ^19^, ^31^ Given Nepetin's diverse biological effects of Nepetin, we thus examined whether Nepetin can affect osteoclast formation and bone resorption. In our study, Nepetin was found to potently inhibited osteoclast differentiation and bone resorption in vitro and protected mice from Ti particle–induced calvarial osteolysis in vivo by reducing the number and activity of osteoclasts. Mechanistically, we found Nepetin treatment attenuated TRAF6‐induced activation of the NF‐κB and MAPK signalling pathways. Interestingly, Nepetin also hindered the induction of TRAF6‐mediated autophagy, a highly conserved cellular process involving the bulk degradation via the lysosomes of cytoplasmic components such as dysfunctional organelles and long‐lived proteins.

Osteoclast differentiation and formation induced following the interaction of RANKL to RANK receptor (see Ref. [32] for review). This leads to the recruitment of TRAF6, which leads to the rapid activation of signalling cascades such as MAPK and NF‐κB pathways. TRAF6 then triggers the recruitment and activation of TAK1 which subsequently activates the inhibitory κB kinase (IKK) complex (consisting of IKKα, IKKβ and IKKγ/NEMO). Activation of the IKK complex induces the phosphorylation and proteasomal degradation of inhibitory κB proteins (predominantly IκBα), releasing the p65/p50 NF‐κB heterodimer subunits to translocate to the nucleus to activate gene transcription.^33^ TAK1 also serves as the upstream MAP3K in the activation phosphorylation of downstream MAPK signalling pathways, ERK, JNK and p38.^33^ The early and efficient activation of MAPK and NF‐κB signalling induces the expression of c‐Fos and NFATc1, two transcriptional factors indispensable for osteoclast differentiation and maturation.^34^ Using immunoblot biochemical analyses, we found that Nepetin treatment inhibited the activation of NF‐κB and MAPK signalling pathways induced by RANKL and the subsequent downstream induction of c‐Fos and NFATc1. Further investigation showed that Nepetin down‐regulated the expression of TRAF6 in a dose‐ and time‐dependent manner.

Autophagy has gained widespread attention for its pivotal role in cell physiology and pathology processes. Autophagy is an evolutionarily conserved lysosomal dependent self‐catabolic pathway critical for cell differentiation and development, and generalized maintenance of cellular homeostasis.^35^ It is characterized by sequestration of cytoplasmic contents including long‐lived proteins, and organelle into lysosomal vesicles termed autophagosomes for bulk degradation and subsequently degraded raw products returned to the cytosol for reuse. Recent studies have shown that autophagy is important for bone homeostasis and is involved in the regulation of bone physiology and pathophysiology.^36^, ^37^ Autophagy has been shown to be induced in response to RANKL, with the expression of autophagic proteins such as autophagy‐related (Atg)4B, 5, Atg7 and Atg 12, and the ratio of LC3‐II/LC3‐I elevated during osteoclast differentiation and bone resorption.^21^, ^38^, ^39^ Recently, mice with osteoclast‐specific deletion of Beclin 1, an indispensable protein involved in the induction of autophagy, exhibited much better bone quality when compared to wild‐type.^14^ Arai et al. further showed using in vitro cellular assays that the RANKL induces autophagy during osteoclast differentiation and the loss of Beclin 1 which is required to initiate the autophagic process inhibited osteoclast differentiation. The overexpression of Beclin 1 enhanced RANKL‐induced osteoclast formation.^14^ Interestingly, the authors further demonstrated that the TRAF6‐dependent ubiquitination of Beclin 1 is necessary for RANKL‐induced autophagy and osteoclast differentiation.^14^

Consistent with these findings, we also observed increased TRAF6‐dependent Beclin 1 ubiquitination following RANKL stimulation and that this effect was completely abolished following Nepetin treatment. We further showed that Nepetin treatment dose‐ and time‐dependently down‐regulated the expression of LC3, Atg5 and Atg12, and inhibition of autophagy and autophagosome formation. We confirmed the inhibition of autophagy by examining the expression of TRAF3 which was previously shown to be degraded via the autophagy system in response to RANKL stimulation.^40^ TRAF3 expression was markedly elevated in BMM cells treated with Nepetin. Interestingly, co‐treatment of cells with Nepetin and rapamycin, a well‐established inducer of autophagy and inhibitor of mTOR signalling, rescued the autophagy defect. This suggests that autophagy activation in osteoclasts is complex and alternative pathways can be activated to restore autophagy. However, further in‐depth investigations into this alternative pathway are necessary.

In short, using computational molecular docking analysis we predicted a potential binding pocket for Nepetin in TRAF6 (Figure S1A). Nepetin could potentially bind leading to inhibition of TRAF6‐dependent ubiquitination of Beclin 1 and attenuation of autophagy. Together with the suppression of TRAF6‐induced activation of NF‐κB and MAPK signalling pathways, Nepetin potently inhibited osteoclast differentiation and bone resorption (Figure S1B). We also found that nepotine had little effect on osteoblast formation (Figure S2). Our findings provide evidence for the use of Nepetin in the treatment of osteolysis conditions.

## CONFLICT OF INTERESTS

The authors declare that they have no competing interests.

## AUTHOR CONTRIBUTION

**Binxiang Chu:** Investigation (equal); Methodology (equal); Writing‐original draft (equal); Writing‐review & editing (equal). **Shenao Chen:** Investigation (equal); Methodology (equal). **Xiaohe Zheng:** Investigation (equal); Methodology (equal); Writing‐original draft (equal). **Jiajing Ye:** Data curation (equal); Formal analysis (equal). **Xu Cheng:** Data curation (equal); Formal analysis (equal); Visualization (equal). **Liwei Zhang:** Data curation (equal); Formal analysis (equal). **Di Guo:** Software (equal); Visualization (equal). **Peng Wang:** Software (equal); Validation (equal); Visualization (equal). **Dun Hong:** Conceptualization (equal); Project administration (equal). **Zhenghua Hong:** Conceptualization (equal); Funding acquisition (equal); Project administration (equal); Resources (equal).

## Supporting information

Fig. S1

Fig. S2

## Data Availability

The data that support the findings of this study are available from the corresponding author upon reasonable request.
