# Supplementary figures and images for: Nepetin inhibits osteoclastogenesis by inhibiting RANKL‐induced activation of NF‐κB and MAPK signalling pathway, and autophagy
Source: J Cell Mol Med. 2020 Nov 1;24(24):14366–80. doi: 10.1111/jcmm.16055 (PMC7754000; doi:10.1111/jcmm.16055)

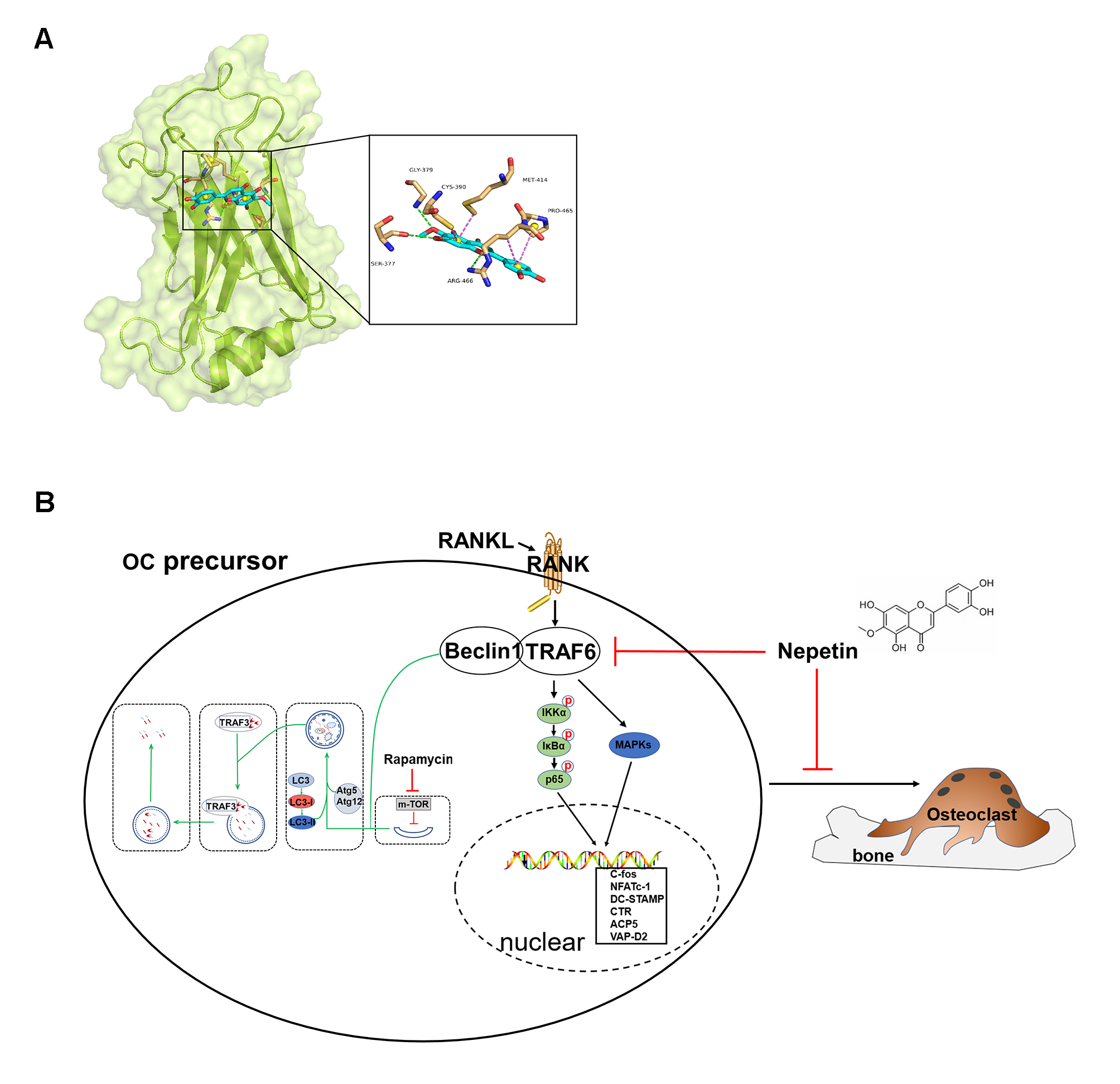

Supplement: Supplementary file 1 — Fig. S1 [file JCMM-24-14366-s001.tif]

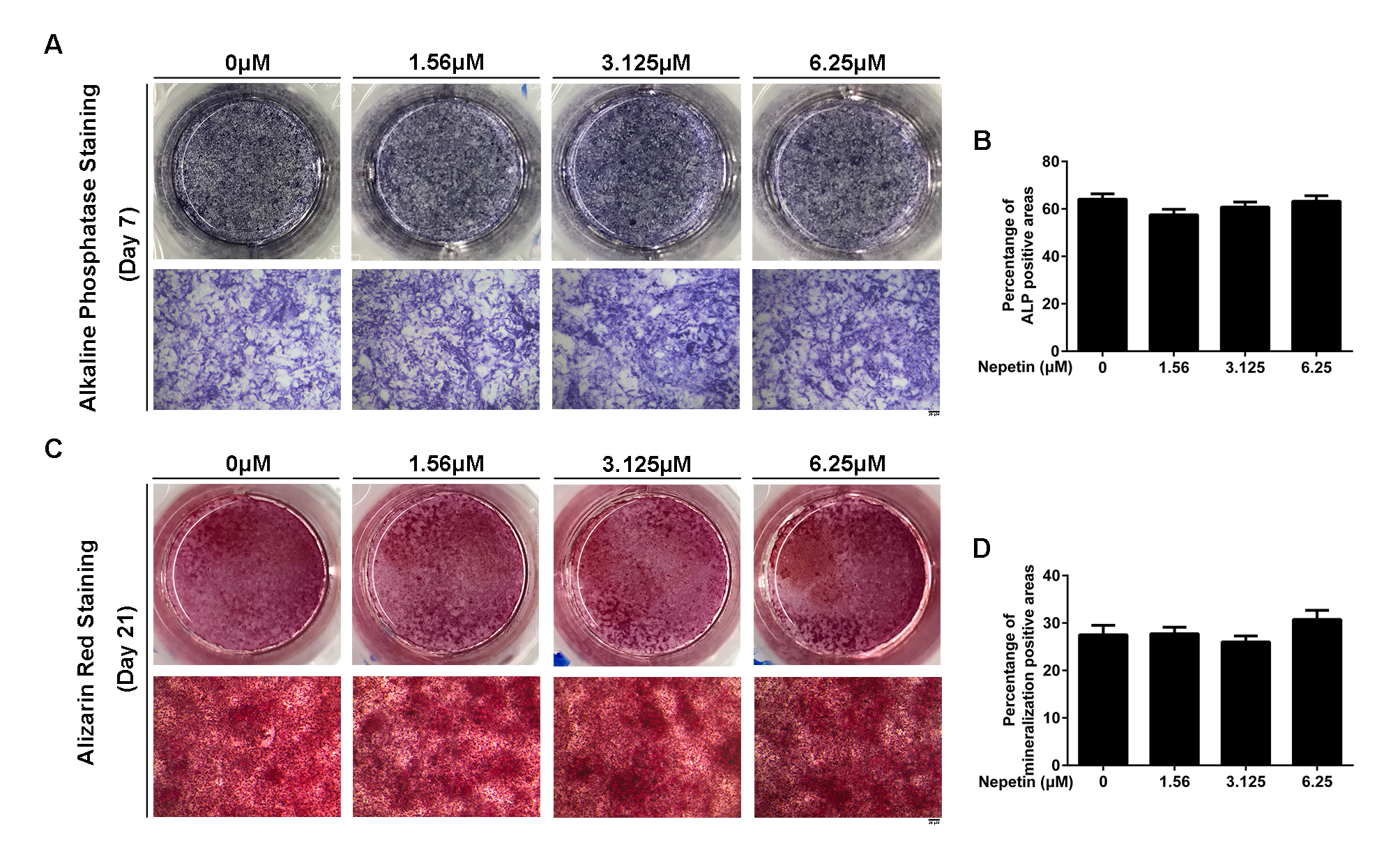

Supplement: Supplementary file 2 — Fig. S2 [file JCMM-24-14366-s002.tif]
